# Supplementary material for: Tumour sampling conditions perturb the metabolic landscape of clear cell renal cell carcinoma
Source: Nat Commun. 2025 Nov 10;16:9896. doi: 10.1038/s41467-025-65676-1 (PMC12603277; doi:10.1038/s41467-025-65676-1)
Supplement: Supplementary file 1 — Supplementary Information [file 41467_2025_65676_MOESM1_ESM.pdf]

Supplementary Information for

Tumour sampling conditions perturb the metabolic landscape of clear cell renal cell carcinoma

Cissy Yong[1,2,3], Christina Schmidt[4], Ming Yang[4], Alexander Von Kriegsheim[5], Anne Y Warren[6], Shubha Anand[7], James N Armitage[1], Antony CP Riddick[1], Thomas J Mitchell[1,2,3], Vishal Patil[8], Kourosh Saeb-Parsy[2], Sakari Vanharanta[9], Grant D Stewart[1,2,3,*], and Christian Frezza [4, 10, *]

[1]Department of Urology, Addenbrooke's Hospital, Cambridge University Hospitals NHS Foundation Trust (CUHFT), Cambridge, UK; [2] Department of Surgery, University of Cambridge, UK; [3] Urological Malignancies Virtual Institute, CRUK Cambridge Centre, University of Cambridge, Cambridge, UK; [4] University of Cologne, Faculty of Medicine and University Hospital Cologne, Institute for Metabolomics in Ageing, Cologne, Germany; [5] Edinburgh Cancer Research UK Centre, Institute of Genetics and Molecular Medicine, UK; [6] Department of Pathology, Addenbrooke's Hospital, CUHFT, UK; [7] Cancer Molecular Diagnostics Laboratory, CRUK Cambridge Cancer Centre, Cambridge, UK; [8] Department of Anaesthetics, Addenbrooke's Hospital, CUHFT, Cambridge, UK; [9] Translational Cancer Medicine Program, Faculty of Medicine, University of Helsinki, Helsinki, Finland; [10] Faculty of Mathematics and Natural Sciences, Institute of Genetics, Cluster of Excellence Cellular Stress Responses in Aging-associated Diseases (CECAD), Cologne, Germany; [*]These authors jointly supervised this work

Corresponding author: Christian Frezza, christian.frezza@uni-koeln.de

Supplementary Fig. 1

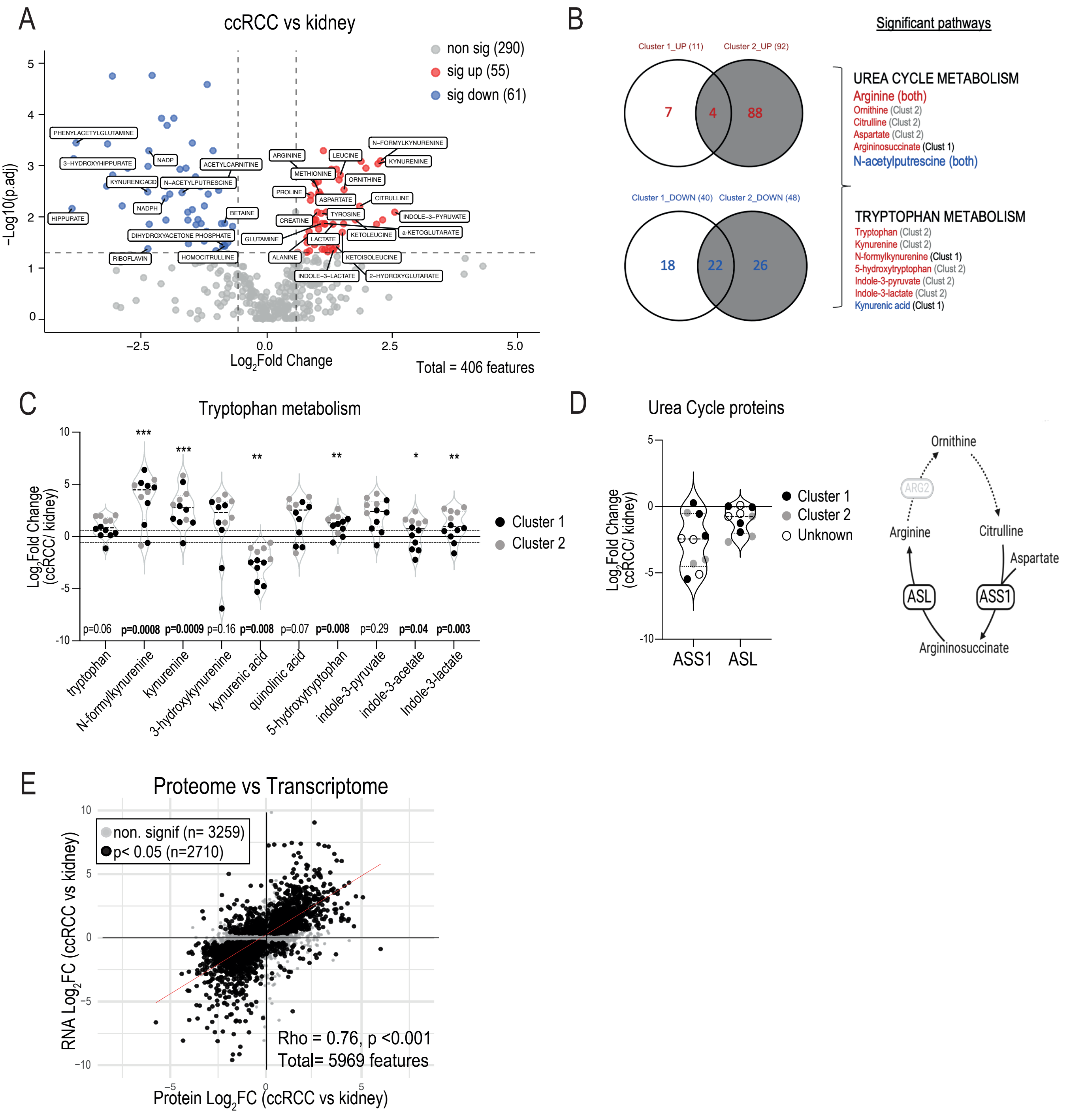

Supplementary Fig. 1: Additional multimodal features of ccRCC tumours.

A. Volcano plot illustrating the metabolic differences between ccRCC tumours vs kidney tissues (n=5 patients). Symbol colour denotes significantly up (red), significantly down (blue), and non-significant scores (grey).

B. Venn diagrams of the significantly accumulated (UP) and decreased (DOWN) metabolites in Cluster 1 and 2 (Fig. 1D). The Urea cycle and Tryptophan pathway detected from Cluster 2 Pathway Analysis are listed with the detected metabolites and the cluster(s) they were significantly detected in. n = 5 patients with 1- 3 multiregional samples per patient tissue, see Supplementary Table 1.

C. Violin plot of the tryptophan pathway metabolite Log2FC (ccRCC vs kidney) with assigned clusters (colour-coded).

D. Violin plot of the protein expression levels for ASS1 and ASL enzymes in ccRCC vs kidney with assigned metabolic clusters (colour-coded). Schematic of the urea cycle pathway.

E. Correlation plot comparing protein and RNA expression of the Log2FC (ccRCC vs kidney). Symbol colour denotes p-value significance.

*p < 0.05, **p < 0.01, ***p<0.001 by two-sided Student's t-test using Benjamini-Hochberg correction (A, C, and D). Correlation coefficients calculated using two-sided Spearman's method with LOESS regression line fitted (E). Source data are provided as a Source Data file. (D) created in BioRender. Burge, S. (2025) https://BioRen-
[illegible]

Supplementary Fig. 2

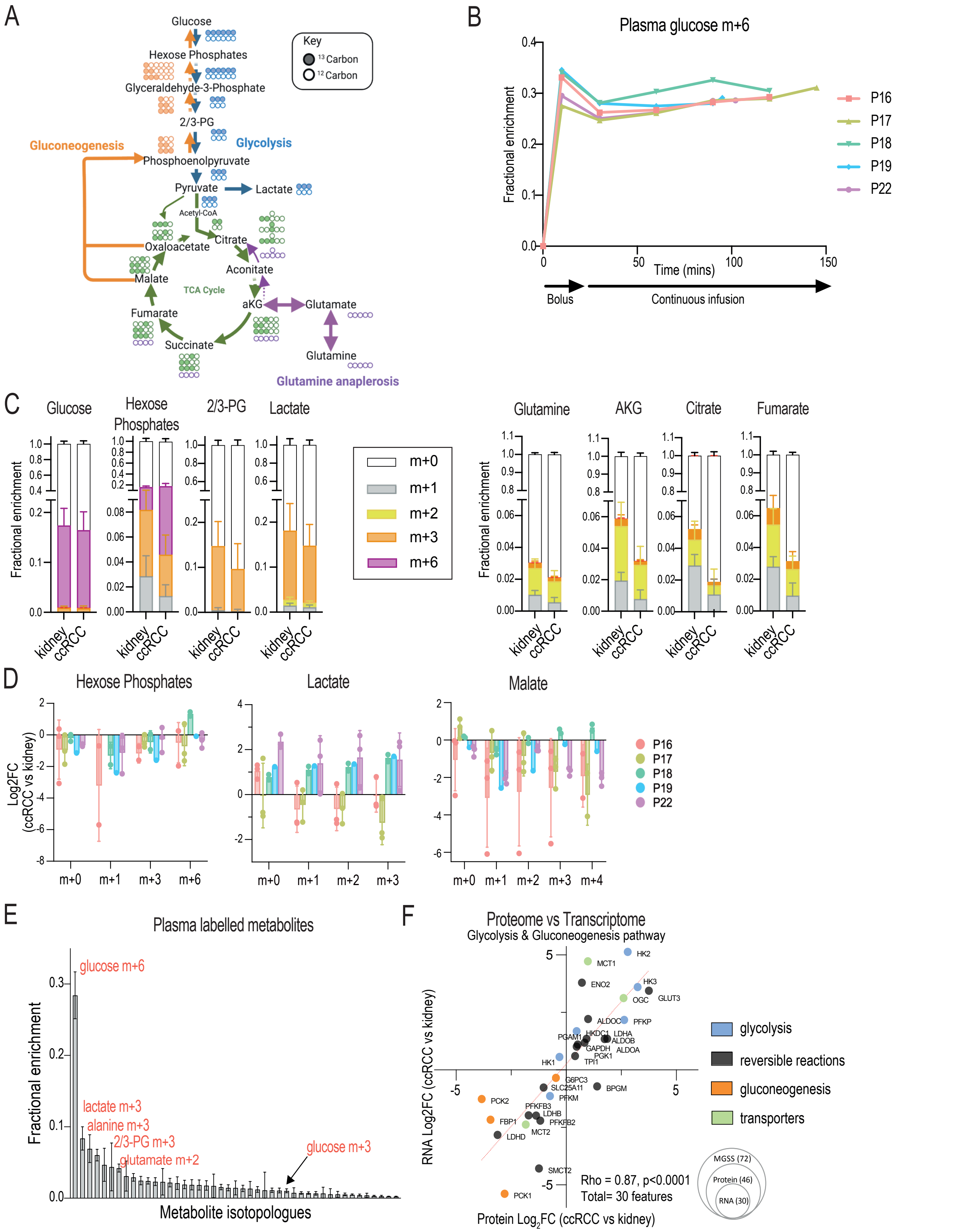

Supplementary Fig. 2: 13C-labelling and multimodal characteristics of 13C6-glucose infusions in patient serum and tissues with ccRCC.

A. Schematic of the major central carbon metabolism pathways displaying the downstream isotopologue (labelling) patterns from 13C6-glucose (colour-coded) infusions.
B. Plasma glucose m+6 enrichment curves of patients (colour-coded, n=5) during the infusion, blood glucose concentrations at time of tissue sampling are shown in Table 1.
C. Proportion of the total tissue pool (Fractional enrichment) of m+n ((colour-coded) for indicated central carbon metabolites.
D. Patient (colour-coded) isotopologue profiles of the indicated central carbon metabolite Log2FC (ccRCC vs kidney). n = 5 patients (symbol shape) with 1- 3 multiregional samples per patient tissue, see Supplementary Table 1.
E. Fractional enrichment of labelled metabolites detected in patients' plasma at time of tissue sampling.
F. Correlation plot comparing protein and RNA expressions of the Log2FC (ccRCC vs kidney) for indicated features from the MGSS glycolysis & gluconeogenesis pathway. Circle map denotes the no. of features in the MGSS and the no. of those features detected in the proteome and transcriptome. Symbol colour denotes metabolic function category.

Data are mean ± SD. Correlation coefficients calculated using two-sided Spearman's method with LOESS regression line fitted (E). Source data are provided as a Source Data file. (A) created in BioRender. Burge, S. (2025) https://BioRender.com/n43g446.

Supplementary Fig. 3

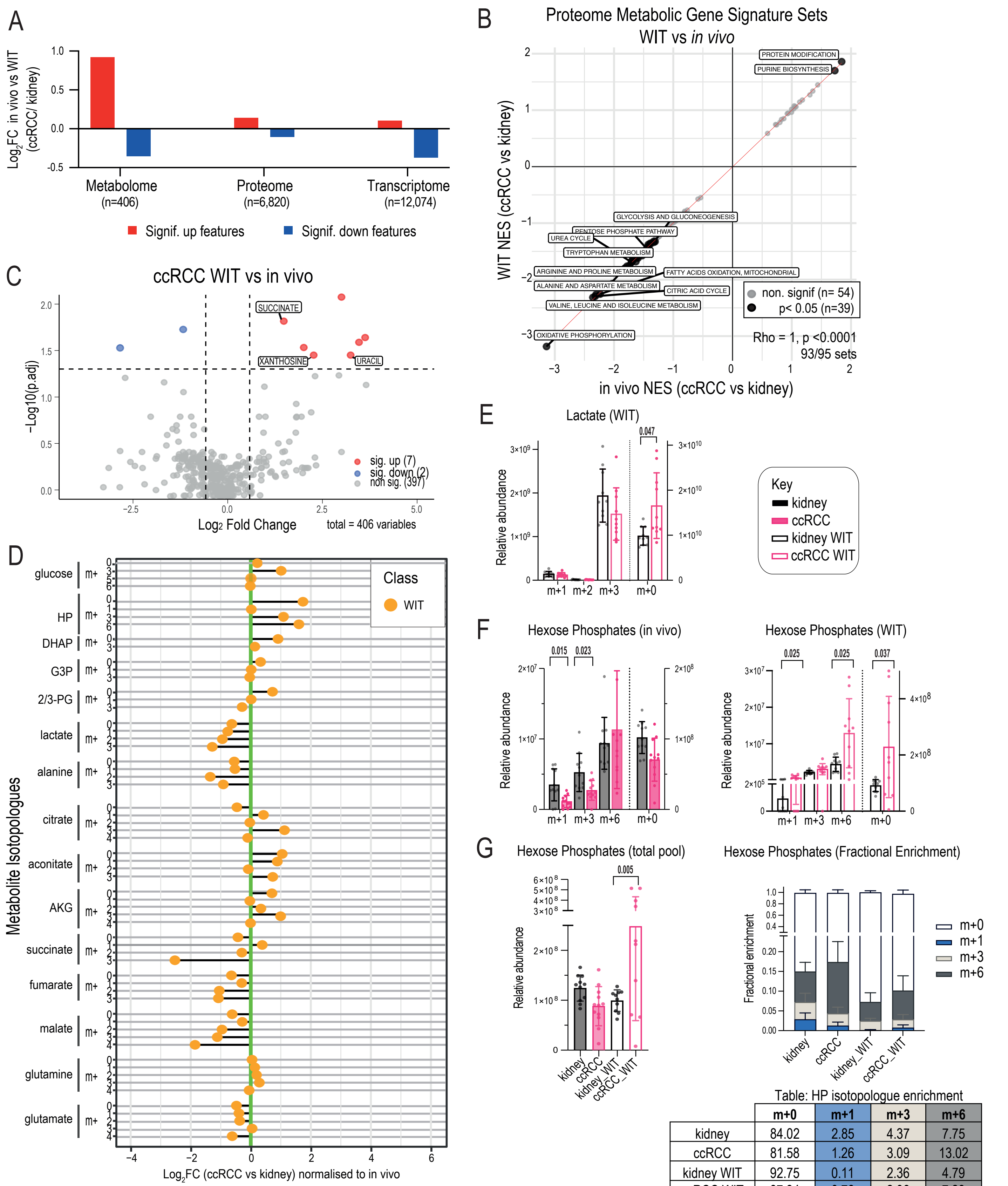

Table: HP isotopologue enrichment

	m+0	m+1	m+3	m+6
kidney	84.02	2.85	4.37	7.75
ccRCC	81.58	1.26	3.09	13.02
kidney WIT	92.75	0.11	2.36	4.79
ccRCC WIT	87.34	0.76	2.06	7.29

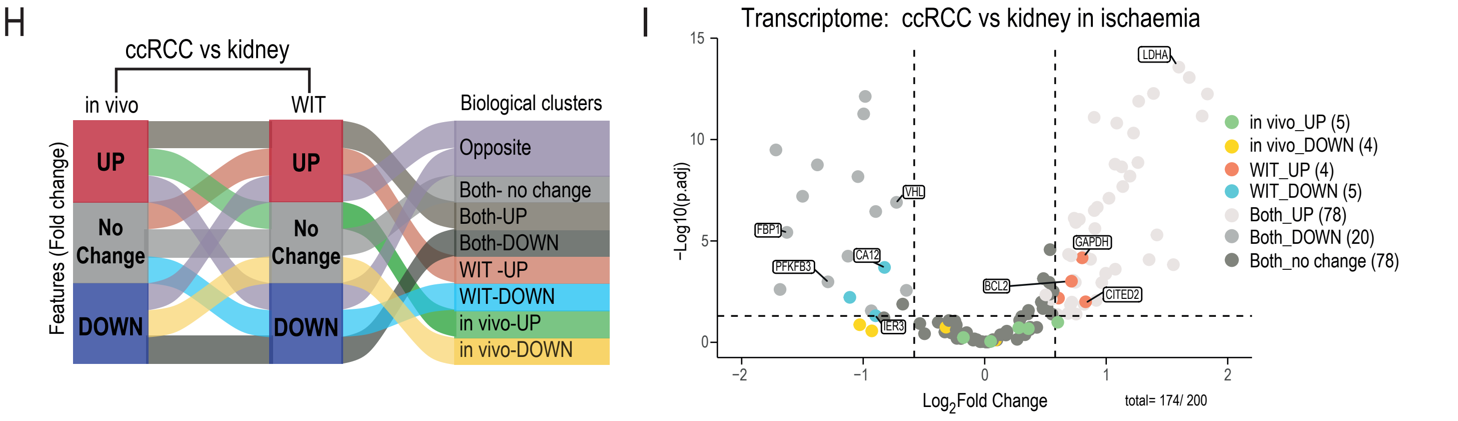

Supplementary Fig. 3: 13C6-labelling and multimodal features of ccRCC tumours exposed to post-operative ischaemia.

A. Bar graph comparing the Log2FC (ccRCC vs kidney) of the no. of significant features (colour-coded) detected in warm ischaemia time (WIT) compared to in vivo in the indicated omics datasets.

B. Correlation plot comparing protein in vivo and WIT NES for the MGSS GSEA comparing ccRCC vs kidney. Symbol colour denotes p-value significance.

C. Volcano plot illustrating the metabolic differences between ccRCC samples (n=5 patients) in in vivo and WIT conditions. Symbol colour denotes significantly up (red), significantly down (blue), and non-significant scores (grey).

D. Dot plot overview of the metabolite isotopologue expressions of the Log2FC (ccRCC vs kidney) comparing WIT (orange) normalised to in vivo conditions (green).

E. Metabolite isotopologue levels of lactate comparing ccRCC (pink) and kidney (black) samples (n=5 patients) taken in WIT conditions (unshaded).

F. Metabolite isotopologue levels of hexose phosphates comparing ccRCC (pink) and kidney (black) samples (n=5 patients) taken in vivo (shaded) and in WIT (unshaded) conditions.

G. Total pool of hexose phosphates and fractional enrichment of hexose phosphates isotopologues with corresponding data in the table, comparing ccRCC (pink) and kidney (black) samples (n=5 patients) taken in vivo (shaded) and in WIT (unshaded) conditions. Barchart (right side) colour denotes specific hexose phosphates isotopologue.

H. Alluvial plot showing the categorising of features into 'biological clusters' based on the fold change (ccRCC vs kidney) between conditions (in vivo and WIT) based on the SiRCle model.

I. Volcano plot of the Hallmark Hypoxia gene set features detected in the transcriptome comparing ischaemic ccRCC vs ischaemic kidneys, colour-coded based on the biological cluster classification (Supplementary Data Fig. 3G).

Correlation coefficients calculated using two-sided Spearman's method with LOESS regression line fitted (B). *p < 0.05, **p < 0.01 by two-sided Student's t-test (C,E, and F) or by one-way ANOVA (G) using Benjamini-Hochberg correction. Threshold for categorisation was a fold change ratio >=1.5 or <=0.67 with adjusted p-value < 0.05 (I). Data are mean ± SD. HP = hexose phosphates; WIT = warm ischaemia time. Source data are provided as a Source Data file.

Supplementary Fig. 4

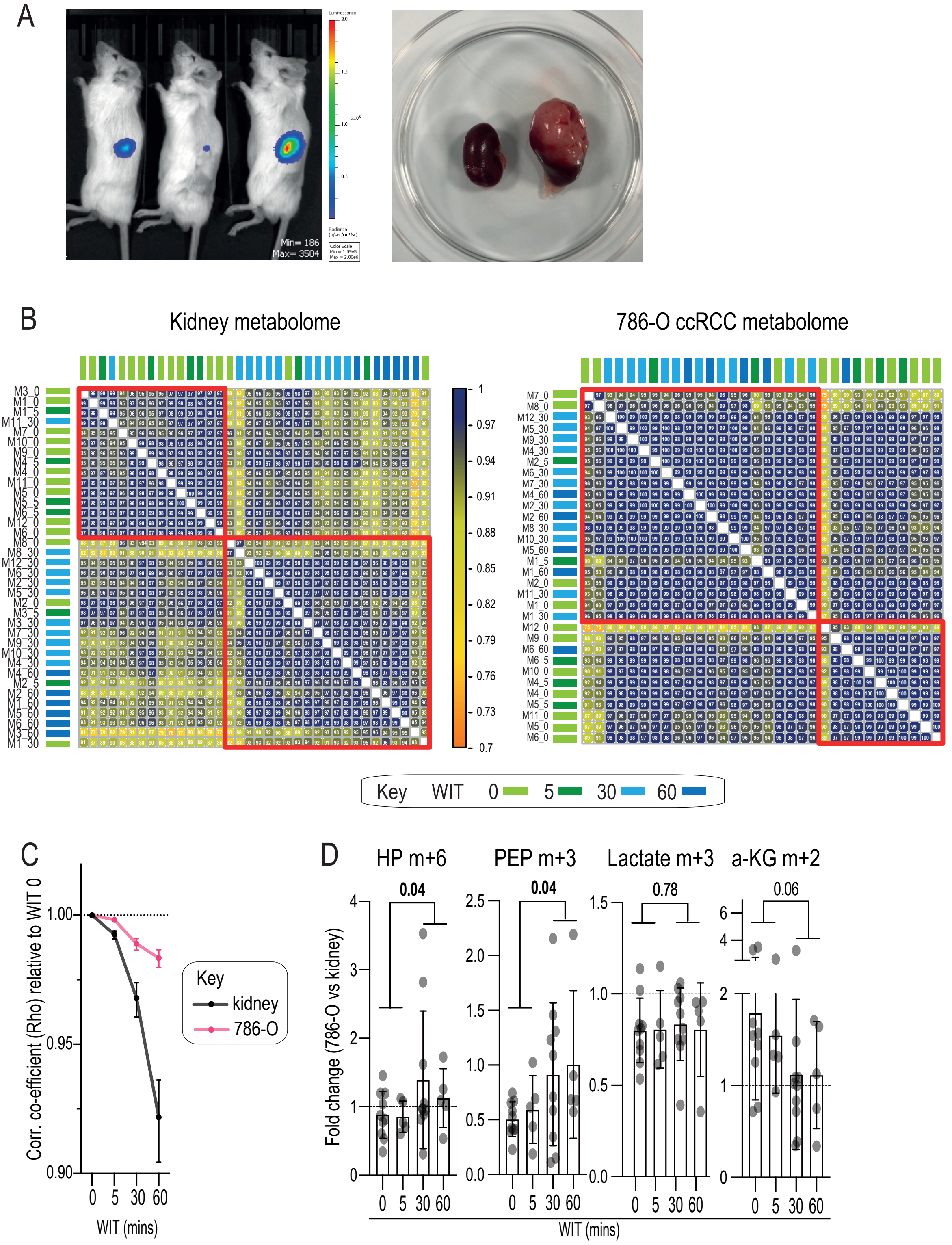

Supplementary Fig. 4: $^{13}C_6$-labelling and metabolomic features of orthotopic 786-O ccRCC xenografts exposed to various durations of warm ischaemia.

A. Bioluminescence image of 786-O-luciferase expressing ccRCC cells xenografted into NSG mice to monitor tumour growth (left panel). Photo taken of paired mouse kidneys showing a normal right kidney and ccRCC tumour growth in left kidney (right panel).

B. Correlation matrix of the metabolome with hierarchical clustering applied to kidney and 786-O tumour samples. For 786-O tumours, mice WIT 0 mins n=11; WIT 5 mins n=5; WIT 30 mins n=11; and WIT 60 mins n=5. For mouse kidneys, mice WIT 0 mins n=12; WIT 5 mins n=6; WIT 30 mins n=12; and WIT 60 mins n=6. Symbol colour denotes WIT time.

C. Correlation graph of tissue-specific (colour-coded) metabolomes at the indicated WIT relative to no ischaemia (WIT 0).

D. Metabolite isotopologue expressions of the fold change (786-O vs kidney) of the indicated metabolites across all WIT timepoints.

Correlation coefficients calculated using two-sided Spearman's method with hierarchal clustering applied (B). *$p < 0.05$ by two-sided Student's t-test using

Supplementary Fig. 5

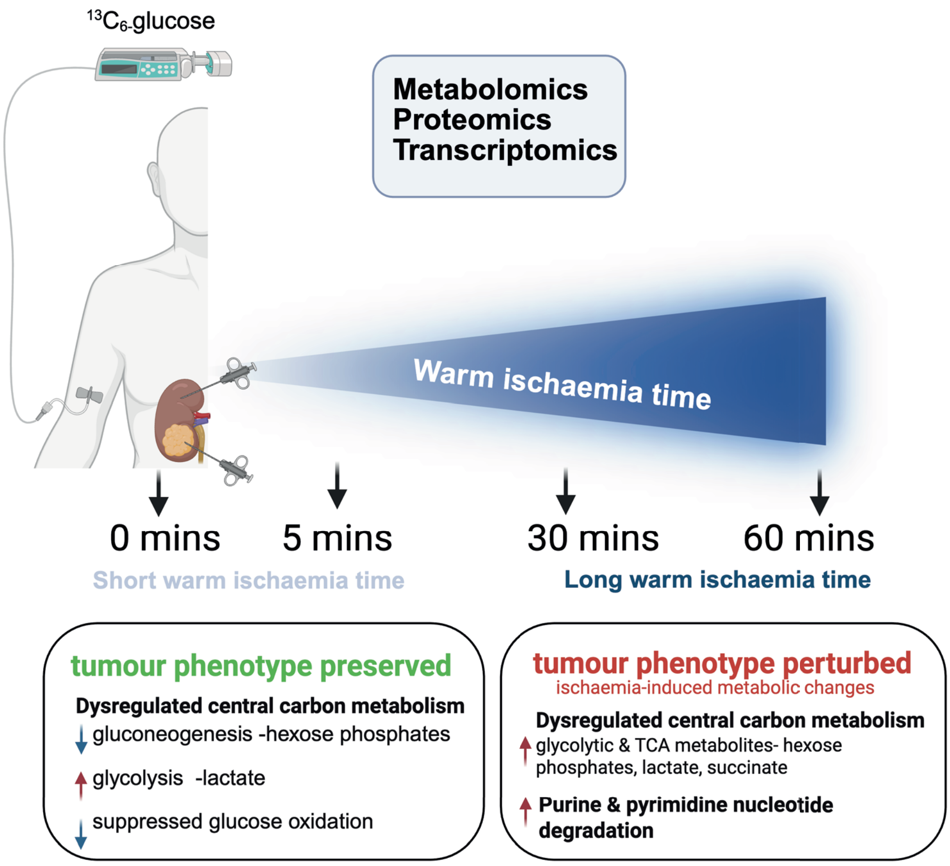

Supplementary Fig. 5: Schematic summary of the impact of warm ischaemia time on tumour metabolic profiling. Schematic created in BioRender. Burge, S. (2025) https://BioRender.com/n43g446.

Supplementary Table 1

ID	Age	Sex	Tumour grade	Tumour size (mm)	Tumour stage (p)	Nodal stage	Metasta-ses stage	VHL sta-tus	Warm Ischae-mia Time (mins)	Kidney samples (in vivo)	ccRCC samples (in vivo)	Kidney sample (WIT)	ccRCC samples (WIT)
P16	70-80	M	4	100	T3a	N0	M0	mutant	60	3	3	3	3
P17	60-70	M	4	125	T4	N1	M0	mutant	45	1	3	2	2
P18	70-80	M	4	160	T3a	N0	M1	mutant	60	2	2	1	1
P19	60-70	M	3	80	T3a	N0	M0	mutant	15	2	1	2	2
P22	50-60	M	4	110	T3a	N0	M0	mutant	10	3	3	2	2

Supplementary Table 1. Patient demvographics and tissue sampling details.

Supplementary Table 2

Gene ID	IV_ log2FC	IV_ Padj	WIT_ log2FC	WIT_ Padj	Biological Cluster
A1BG	2.11389262	0.00460557	1.78995476	0.01850410	Both_UP
A1CF	-0.46447483	0.10691627	-0.14780442	0.25802401	Both_no change
A2M	1.15536265	0.00157648	0.98207477	0.00051213	Both_UP
AAAS	0.12612138	0.05839733	-0.12180874	0.08595429	Both_no change
AACS	-1.06774031	7.87E-09	-0.71719253	0.00039992	Both_DOWN
AADAT	-2.54759636	0.00020108	-2.57730764	1.02E-07	Both_DOWN
AAK1	-0.54898886	0.00020124	-0.51636034	2.54E-05	Both_no change
AAMDC	-0.34009981	0.00078175	-0.08422009	0.20582532	Both_no change
AAMP	-0.13713688	0.02925615	-0.07690399	0.00121959	Both_no change
AAR2	0.18189417	0.05192463	-0.41341222	0.01132827	Both_no change
AARS1	0.42421918	0.01788763	0.30036741	0.01153606	Both_no change
AARS2	-0.37198078	0.01399091	-0.60595311	0.00306613	Both_DOWN
AASDHPPT	0.05700535	0.15958964	0.16530943	0.01036517	Both_no change
AASS	-2.48312248	3.51E-08	-2.69952239	1.75E-08	Both_DOWN
ABAT	-3.29962597	2.45E-08	-3.63344007	8.70E-11	Both_DOWN
ABCA6	-1.74626711	5.62E-08	-1.00027878	0.08273514	IV_DOWN
ABCA8	-2.44784004	1.15E-08	-3.33475067	1.17E-06	Both_DOWN
ABCB1	-1.80750972	1.41E-06	-2.43915425	4.01E-09	Both_DOWN
ABCB6	1.21682244	0.02357443	0.20913260	0.02817008	Both_UP
ABCB7	-0.30442592	0.19244824	-1.05930638	4.53E-05	WIT_DOWN
ABCB8	-1.37734407	3.65E-06	-2.23078975	7.87E-11	Both_DOWN
ABCB9	2.76040805	0.00992937	0.64311073	0.00229490	Both_UP
ABCC1	0.97699245	0.01098862	0.28070088	0.00039730	Both_UP
ABCC2	-1.64810941	0.00022803	-1.93770668	6.96E-07	Both_DOWN
ABCC3	1.45665737	0.00280635	0.90923689	1.68E-05	Both_UP
ABCC4	-0.83969033		-1.05274409	9.28E-09	Both_DOWN
ABCC6	-1.10856120	0.00269405	-1.48132641	1.88E-05	Both_DOWN
ABCD1	0.99211299	0.00049909	0.96517172	0.00043455	Both_UP
ABCD3	-0.80278726	0.00032606	-1.50939669	8.89E-06	Both_DOWN
ABCE1	0.16806880	0.11030352	0.07421695	0.12083120	Both_no change
ABCF1	-0.26630354	0.00013981	0.08642590	0.06966274	Both_no change
ABCF2	-0.12089196	0.01876405	0.04337863	0.21615723	Both_no change

Supplementary Table 2: Evaluating the proteome (ccRCC vs kidney) between ischaemia and in vivo (IV) conditions using the SiRCle model. Two-sided Student's t-test using Benjamini-Hochberg correction was used. For full table results see source data file.
