# Supplementary material for: Tumour sampling conditions perturb the metabolic landscape of clear cell renal cell carcinoma
Source: Nat Commun. 2025 Nov 10;16:9896. doi: 10.1038/s41467-025-65676-1 (PMC12603277; doi:10.1038/s41467-025-65676-1)
Supplement: Supplementary file 2 — Reporting Summary [file 41467_2025_65676_MOESM2_ESM.pdf]

nature portfolio

Corresponding author(s): Prof. Christian Frezza

Last updated by author(s): Sep 26, 2025

Reporting Summary

Nature Portfolio wishes to improve the reproducibility of the work that we publish. This form provides structure for consistency and transparency in reporting. For further information on Nature Portfolio policies, see our Editorial Policies and the Editorial Policy Checklist.

Statistics

For all statistical analyses, confirm that the following items are present in the figure legend, table legend, main text, or Methods section.

n/a	Confirmed	
☐	☒	The exact sample size (*n*) for each experimental group/condition, given as a discrete number and unit of measurement
☐	☒	A statement on whether measurements were taken from distinct samples or whether the same sample was measured repeatedly
☐	☒	The statistical test(s) used AND whether they are one- or two-sided<br>*Only common tests should be described solely by name; describe more complex techniques in the Methods section.*
☐	☒	A description of all covariates tested
☐	☒	A description of any assumptions or corrections, such as tests of normality and adjustment for multiple comparisons
☐	☒	A full description of the statistical parameters including central tendency (e.g. means) or other basic estimates (e.g. regression coefficient) AND variation (e.g. standard deviation) or associated estimates of uncertainty (e.g. confidence intervals)
☐	☒	For null hypothesis testing, the test statistic (e.g. *F*, *t*, *r*) with confidence intervals, effect sizes, degrees of freedom and *P* value noted<br>*Give P values as exact values whenever suitable.*
☒	☐	For Bayesian analysis, information on the choice of priors and Markov chain Monte Carlo settings
☐	☒	For hierarchical and complex designs, identification of the appropriate level for tests and full reporting of outcomes
☐	☒	Estimates of effect sizes (e.g. Cohen's *d*, Pearson's *r*), indicating how they were calculated

Our web collection on statistics for biologists contains articles on many of the points above.

Software and code

Policy information about availability of computer code

Data collection	LC/MS data was collected using a Q Exactive Hydrid Quadrupole-Orbitrap Mass spectrometer (Thermo Scientific, USA) coupled to a Dionex Ultimate 3000 UHPLC (Dionex, USA). Proteomics data was collected using an Orbitrap Fusion Lumos mass spectrometer (ThermoFisher Scientific, USA). RNA sequencing data was collected using a NextSeq 500 (Illumina). Gene panel data was collected using a Nextseq 2000 (Illumina). Bioluminescence data was collected using an IVIS® spectrum (PerkinElmer, USA)
Data analysis	Graphpad Prism v9.0.1, Metaboanalyst v5.0, and R studio v4.1.2 were used for data analysis. Metabolomics data was analysed using Tracefinder 5.0 software (Thermo Fisher, USA). Proteomics data was analysed using Spectronaut 15 pipeline (Biognosis, Switzerland) using directDIA against the Uniprot Human database. RNA seq data were mapped to the genome using STARmapper (v2.7.153) and Ensembl Homo Sapiens GRCh38 (release 105) reference genome. QC of the reads was performed using FastQC v0.11.4 and were trimmed using TrimGalore v0.5.0. Base calling after sequencing was done using Illumina bcl2gastq software. Batch correction was performed using the COMBAT_seq correction method from the SVA package55. Normalisation and differential gene expression analysis was performed using the edgeR package v3.26.556. Gene set enrichment analyses was performed using the GSEA software v4.2.2 (Broad Institute).Gene panel analysis was analysed using the in-house Cancer Molecular Diagnostics Laboratory (CRUK, Cambridge, UK) GCP compliant pipeline (version 0.41) which utilised the following main algorithms: bwa-mem (hg38 alt contig aware alignment), samtools (PCR Duplicates removal), GATK (Base Quality Score, Recalibration, InDel Realignment), GATK's Haplotype Caller (Germline Variant Calling), GATK's MuTect2 (Somatic Variant Calling), GATK (CNV Calling), Delly (Non-CNV SV Calling), and Annovar (Variant Annotation). Bioluminescene data was analysed using Living Image software (PerkinElmer, USA). Adobe Illustrator V28.7.1 and BioRender software (https://app.biorender.com/) was used to create paper schematics and figures. All code has been previously published and is referenced appropriately in the Methods section of the paper.

For manuscripts utilizing custom algorithms or software that are central to the research but not yet described in published literature, software must be made available to editors and reviewers. We strongly encourage code deposition in a community repository (e.g. GitHub). See the Nature Portfolio guidelines for submitting code & software for further information.

Data

Policy information about availability of data

All manuscripts must include a data availability statement. This statement should provide the following information, where applicable:

- Accession codes, unique identifiers, or web links for publicly available datasets
- A description of any restrictions on data availability
- For clinical datasets or third party data, please ensure that the statement adheres to our policy

The mass spectrometry proteomics data have been deposited to the ProteomeXchange Consortium via the PRIDE partner repository with the dataset identifier PXD054588 (https://proteomecentral.proteomexchange.org/cgi/GetDataset?ID=PXD054588). The RNA sequencing data has been deposited to the Gene Expression Omnibus (GEO) data repository with the accession no: GSE274774 (https://www.ncbi.nlm.nih.gov/geo/query/acc.cgi?acc=GSE274774). The metabolomics data has been deposited to the Metabolomics Workbench repository with the study ID: ST004207 (http://dx.doi.org/10.21228/M8VC26). Source data are provided with this paper.

Research involving human participants, their data, or biological material

Policy information about studies with human participants or human data. See also policy information about sex, gender (identity/presentation), and sexual orientation and race, ethnicity and racism.

Reporting on sex and gender	Sex was determined based on assignment as per clinical data records with consent obtained from patients. Sex-based analysis was not performed in this study due to the small sample size (n=5) and lack of relevance to the hypothesis of this study i.e. do tissue sampling conditions perturb the metabolic and molecular landscape of ccRCC tumours.
Reporting on race, ethnicity, or other socially relevant groupings	This study did not report on these characteristics.
Population characteristics	Age, sex, histology, pathology staging, tumour grading, VHL status, treatment category (i.e. surgical approach)
Recruitment	Adults were identified and enrolled into this study from uro-oncology multidisciplinary team meetings (MDT) and/ or renal oncology clinics by the clinical team at Addenbrooke's Hospital, CUHFT following informed consent from all participants. Eligible patients were further discussed with the operating surgeon and the anaesthetic study lead to ensure suitability of patients for labelled-glucose infusions and intraoperative sampling. Eligibility criteria included adults (aged 18 years and over) diagnosed with a 'renal mass' undergoing a nephrectomy as part of their clinical management at CUHFT.<br>As this study used a novel intraoperative sampling technique to sample perfused tissues as well as to assess the impact of ischaemia on tissue sampling, we intentionally recruited patients with ccRCC undergoing open surgery (compared to laparoscopic or robotic surgery). Firstly, this minimises any potential ischaemia time between surgery and conventional research tissue sampling, and secondly, this surgical approach allows optimal control of intraoperative bleeding (due to risk of intraoperative sampling). This has lead to the sampling of tumours of a similar advanced stage and high grade which likely impacts the generalisability of some of our findings, as discussed in the limitations.
Ethics oversight	UK Human Research Authority, East of England- Cambridge Central Research Ethics Committee (Ref: 19/EE/0161).

Note that full information on the approval of the study protocol must also be provided in the manuscript.

Field-specific reporting

Please select the one below that is the best fit for your research. If you are not sure, read the appropriate sections before making your selection.

[x] Life sciences [ ] Behavioural & social sciences [ ] Ecological, evolutionary & environmental sciences

For a reference copy of the document with all sections, see nature.com/documents/nr-reporting-summary-flat.pdf

Life sciences study design

All studies must disclose on these points even when the disclosure is negative.

Sample size	No sample-size calculation was performed. Sample size was chosen based on a pragmatic approach to patient recruitment and isotopic tracer resource availability as well as previous sample sizes for similar experiments based on previous literature. The patient sample size was sufficient to deduce a difference between tissue sampling groups (in vivo vs ischaemia), which was then further evaluated in our mouse model. No sample-size calculation was performed for mouse experiments, however a minimum of 5 to a maximum of 12 biological replicates (individual mice) were used for each treatment group, which is in keeping with previous sample sizes for similar experiments based on the literature and was sufficient to deduce a statistically significant difference between treatment groups exposed to varying warm ischaemic times.
Data exclusions	For isotopologue analysis by LC/MS, peak areas that could not be confidently assessed as the identified metabolite were excluded. For RNA seq analysis, reads with a poor mapping quality (< 10), mapping to multiple loci, or to overlapping gene regions were excluded to avoid ambiguity and false positives.
Replication	All mouse experiments were performed across independent experiments with at least 3 biological replicates of786-O ccRCC cells. Each biological replicate was defined as an independent culture of cells. All replication efforts for mouse and in vitro experiment were successful.

	Patient samples were collected from individuals on the day of their surgery over the course of one year. Multi-regional tissue sampling, where possible, was taken from individual patients and treated as biological replicates. Human sample studies were not replicated from individual patients as tissues can only be sampled at one time point.
Randomization	Randomization was not relevant to this study as patients did not undergo an intervention requiring randomisation. Mice were randomly allocated for injections.
Blinding	Blinding of participants was not relevant as all patients received the same intervention and isotopic tracer. All samples were given a de-identified code during data collection for mass spectrometry analysis, RNA-sequencing, and gene panel analysis.

Reporting for specific materials, systems and methods

We require information from authors about some types of materials, experimental systems and methods used in many studies. Here, indicate whether each material, system or method listed is relevant to your study. If you are not sure if a list item applies to your research, read the appropriate section before selecting a response.

Materials & experimental systems

n/a	Involved in the study	
x		Antibodies
	x	Eukaryotic cell lines
x		Palaeontology and archaeology
	x	Animals and other organisms
	x	Clinical data
x		Dual use research of concern
x		Plants

Methods

n/a	Involved in the study	
x		ChIP-seq
x		Flow cytometry
x		MRI-based neuroimaging

Eukaryotic cell lines

Policy information about cell lines and Sex and Gender in Research

Cell line source(s)	786-O cells were obtained from the American Type Culture Collection, ATCC (CRL-1932). Cells were engineered to stably express luciferase as previously described (https://doi.org/10.1038/nm.3029). This cell line was derived from a male patient. This cell was used as it is an established and well characterised VHL-mutant ccRCC cell line. Sex-based analysis was not performed as it was not relevant to the goal of the study which was to assess the impact of ischaemia on the metabolic phenotype of tumours.
Authentication	786-O cells were not authenticated after purchase.
Mycoplasma contamination	Mycoplasma negativity was confirmed by the MycoAlertTM Mycoplasma Detection Kit (Lonza, LT07-318).
Commonly misidentified lines (See ICLAC register)	No commonly misidentified lines were used in this paper.

Animals and other research organisms

Policy information about studies involving animals; ARRIVE guidelines recommended for reporting animal research, and Sex and Gender in Research

Laboratory animals	Healthy male NSG mice (n=5-12) between the ages for 8-13 weeks were used in this study. Mice were purchased from Charles River Laboratories, UK. All mice were housed in specific-pathogen-free animal facilities with ad libitum access to food and water. Prior to the described studies, mice were monitored regularly and determined to be healthy by the veterinary staff.
Wild animals	No wild animals were used in this study.
Reporting on sex	Only male mice were used for this study. Sex-based analysis was not performed as it was not relevant to the goal of the study which was to assess the impact of ischaemia on metabolic analyses.
Field-collected samples	No field collected samples were used in this study.
Ethics oversight	All procedures were approved by the UK Home Office (Project Licence No. PFCB122AA) and the University of Cambridge Animal Welfare and Ethical Review Body. All experiments were conducted in strict accordance with the UK Animals (Scientific Procedures) Act 1986 by personnel with the appropriate personal licence.

Note that full information on the approval of the study protocol must also be provided in the manuscript.

Clinical data

Policy information about clinical studies

All manuscripts should comply with the ICMJE guidelines for publication of clinical research and a completed CONSORT checklist must be included with all submissions.

Clinical trial registration	This study is not registered as a clinical trial. Study ethics was approved by the UK HRA Research Ethics Committee (19/EE/0161).
Study protocol	This study is not registered as a clinical trial.
Data collection	All patients were recruited at Addenbrooke's Hospital, Cambridge University Hospitals, UK, between February- December 2021 with data collected on the day of patient surgery.
Outcomes	Clinical outcome measures are not applicable to this study.

Plants

Seed stocks	N/A
Novel plant genotypes	N/A
Authentication	N/A
